# Supplementary material for: CYCLIN-B1/2 and -D1 act in opposition to coordinate cortical progenitor self-renewal and lineage commitment
Source: Nat Commun. 2020 Jun 9;11:2898. doi: 10.1038/s41467-020-16597-8 (PMC7283355; doi:10.1038/s41467-020-16597-8)

Supplementary Information

CYCLIN-B1/2 and -D1 act in opposition to coordinate cortical progenitor self-renewal and lineage commitment

Daniel W. Hagey et al.

This PDF contains:

Supplementary Figures 1-13

Supplementary Fig. 1 (Hagey et al).

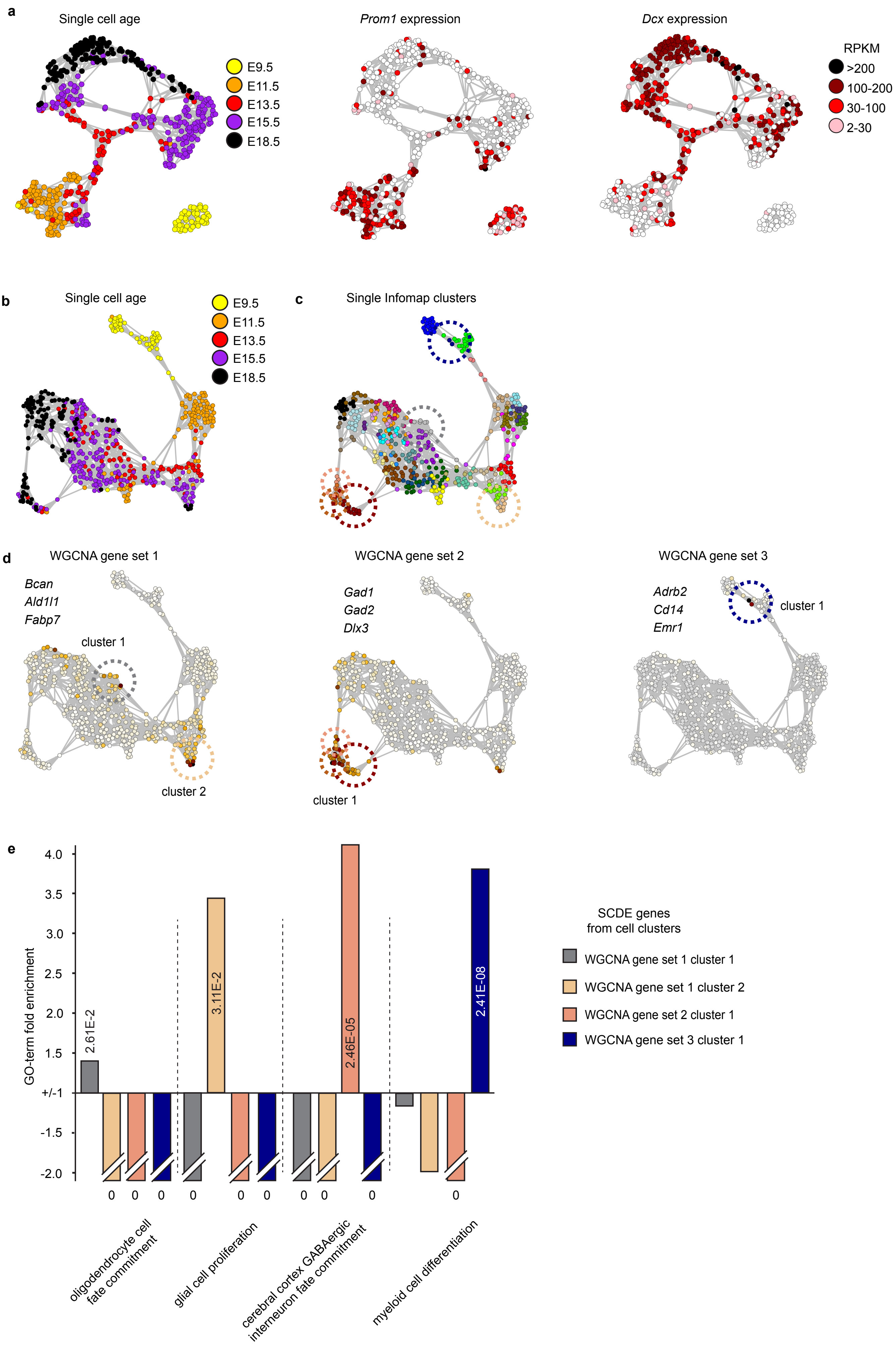

Supplementary Fig. 1 Identification of sequenced cells of non-cortical origins. **a** *t*SNE-NN maps of all sequenced cells passing quality control (QC). The graphs are based on variable genes expressed above 0.5 rpkms and coloured by the age that cells were picked at, or by their expression levels of markers for progenitors (*Prom1*) or neurons (*Dcx*). **b, c** *t*SNE-NN maps instructed by genes specific for astrocytes, oligodendrocytes, interneurons, immune cells, ependymal cells, mural cells and endothelial cells. The graphs are coloured by the age that the cells were picked at (**b**) or by the Infomap cluster colours (**c**). Clusters of different cell types identified in downstream analyses are circled in (**c**). **d** *t*SNE- NN maps coloured by WGCNA enrichment scores for gene sets expressed by glial cells (gene set 1), interneurons (gene set 2) or immune cells (gene set 3), with representative genes for each gene sets presented. Circles correspond to Infomap clusters highlighted in (**c**). **e** GO-term fold enrichments and p-values for genes that were specific to Infomap clusters highlighted in (**c, d**) according to single-cell differential expression (SCDE). P-values for GO-term analysis are derived from a Binominal test.

Supplementary Fig. 2 (Hagey et al).

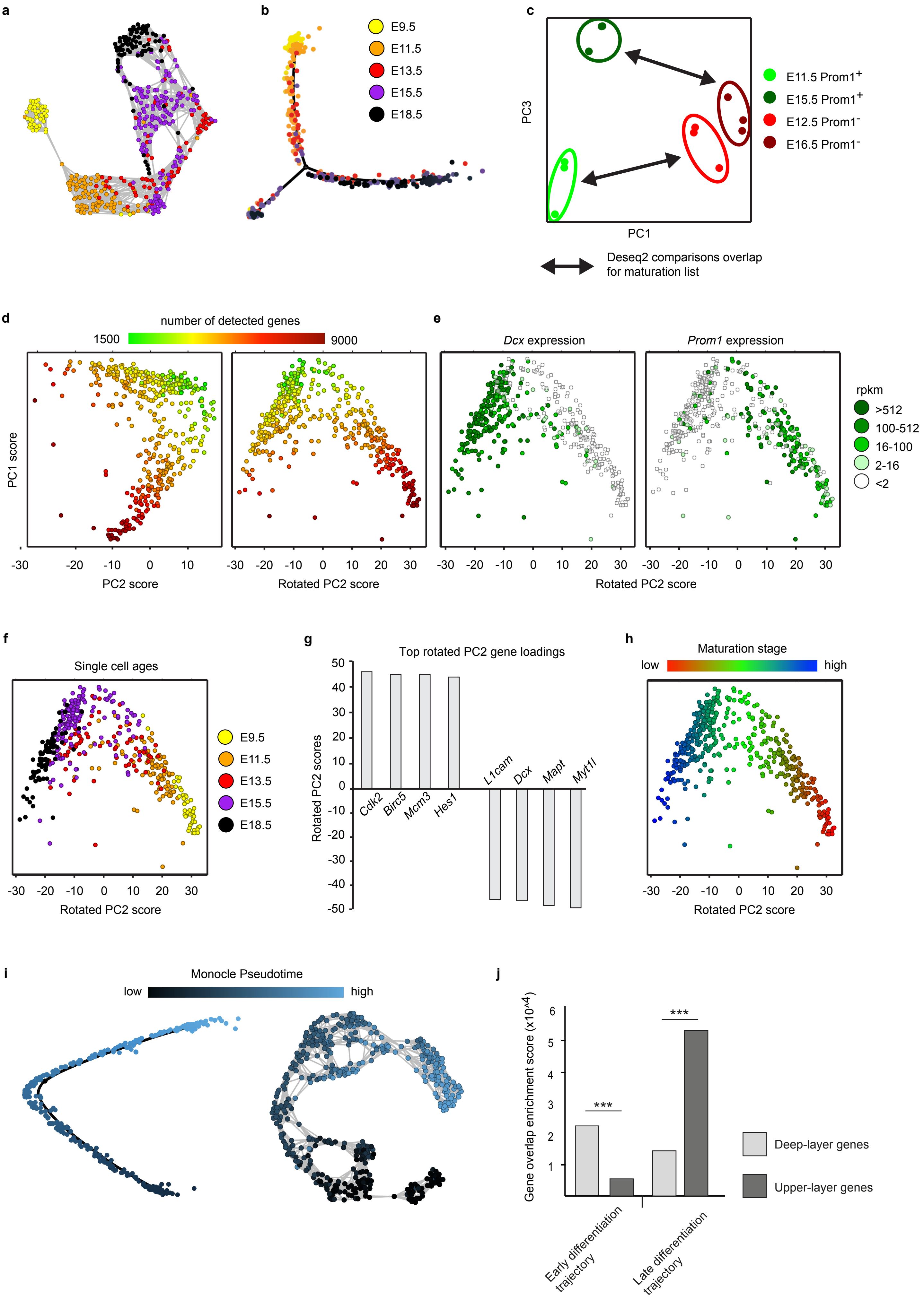

Supplementary Fig. 2 Maturation stage values of sequenced cells. **a, b** Cortical neural cells arranged according to our *t*SNE-NN based method (**a**) or Monocle DDRTree (**b**), informed by previously defined cortical layer identity genes[26]. Colour code denotes embryonic age of analysed cells; E9.5 (yellow), E11.5 (orange), E13.5 (red), E15.5 (purple) and E18.5 (black). **c** PCA on RNA-sequence data derived from E11.5 *Prom1*$^+$, E15.5 *Prom1*$^+$, E12.5 *Prom1*$^-$ and E16.5 *Prom1*$^-$ cortical cells. Arrows illustrate Deseq2 comparisons used to generate lists of genes generally expressed during cortical neurogenesis, which were used to inform the *t*SNE-NN maps presented in (**i**) and the main figures. **d** PCA on cortical single-cell RNA-sequence data, before and after rotation, coloured by the number of genes detected in each cell. **e** Rotated PCA coloured by expression (RPKMs) of *Dcx* or *Prom1*, where dark green denotes high expression and white, low expression. **f** Rotated PCA coloured by the embryonic stage the cells were picked at, as in (**a**). **g** Four of the top ten gene loadings informing rotated PC2 and their loading scores. **h** Rotated PCA coloured by cell's maturation stage values, where red denotes low values and dark blue, high values. **i** Cortical neural cells arranged by Monocle or our *t*SNE-NN map methods, when informed by differentiation genes derived from (**c**) and previously defined cortical layer identity genes[26]. Colour code denotes Monocle Pseudotime values, where dark blue represents a low Pseudotime and light blue, a high Pseudotime. **j** Graph shows the overlap enrichment scores of known deep- and upper-layer neural genes[1] in early and late differentiation trajectories neuroblast gene sets (Supplementary Table 4). P-values for gene expression differences in early- and late differentiation trajectories are 4.61e-9 and 5.94e-3, respectively. Statistics based on Yates Chi-squared tests; *** = $p<0.001$.

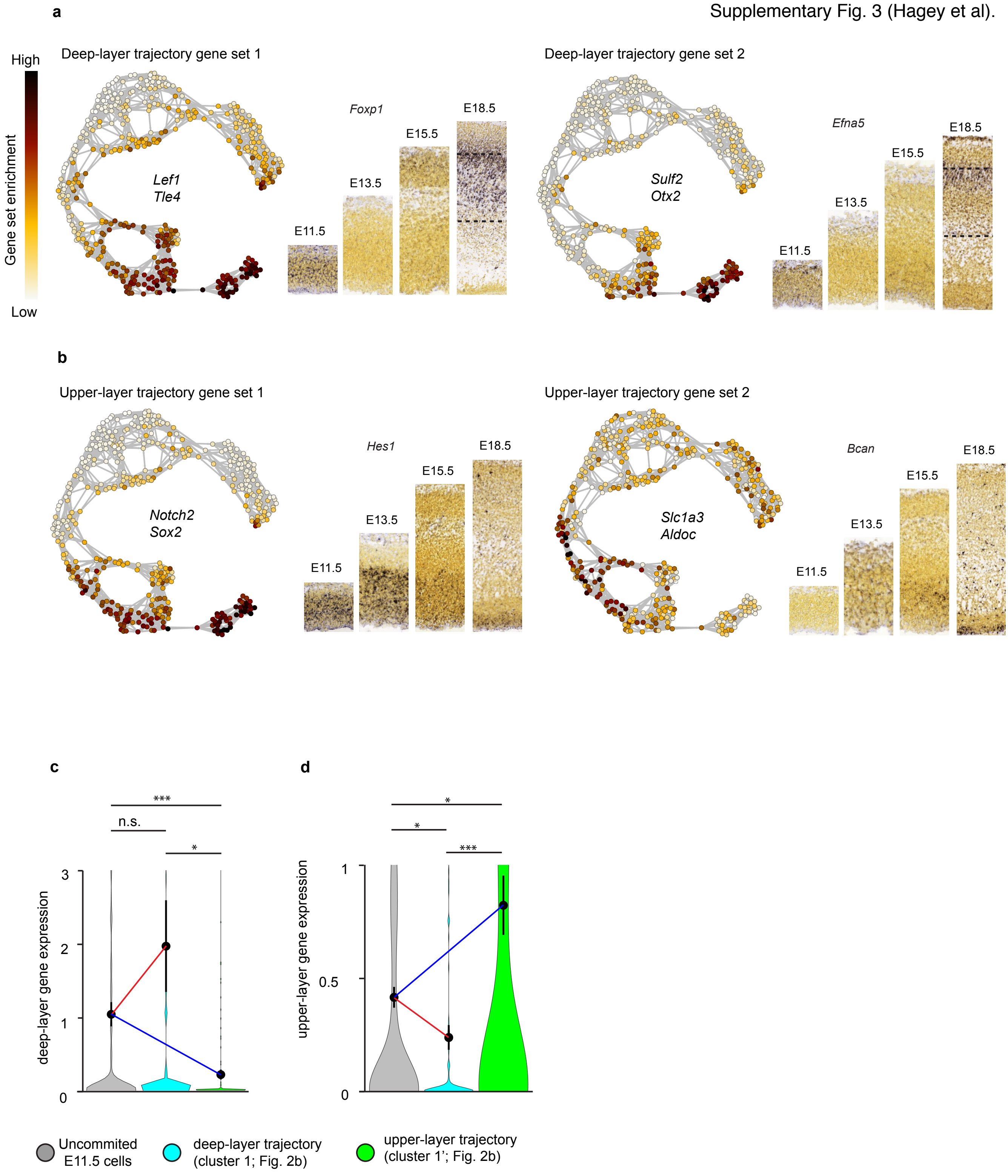
a
Deep-layer trajectory gene set 1
High
Gene set enrichment
Low
Lef1
Tle4
Foxp1
E11.5
E13.5
E15.5
E18.5
Deep-layer trajectory gene set 2
Sulf2
Otx2
Efna5
E11.5
E13.5
E15.5
E18.5
b
Upper-layer trajectory gene set 1
Notch2
Sox2
Hes1
E11.5
E13.5
E15.5
E18.5
Upper-layer trajectory gene set 2
Slc1a3
Aldoc
Bcan
E11.5
E13.5
E15.5
E18.5
c
***
n.s.
*
deep-layer gene expression
0
1
2
3
d
*
*
***
upper-layer gene expression
0
0.5
1
Uncommited E11.5 cells
deep-layer trajectory (cluster 1; Fig. 2b)
upper-layer trajectory (cluster 1'; Fig. 2b)

Supplementary Fig. 3 WGCNA gene sets enriched at early stages of deep- or upper-layer neurogenesis. **a, b** *t*SNE-NN maps displaying two different WGCNA gene sets for the deep-layer trajectory (**a**) and two for the upper-layer trajectory (**b**). The expression of representative genes from each gene set are shown with *in situ* hybridization of E11.5, E13.5, E15.5 and E18.5 cortices. **c, d** Violin plots of average normalized RPKMs for deep- (represented by *Fezf2*, *Tbr1*, *Nr4a3, Dkk3 and Otx2*) or upper-layer genes (represented by *Pou3f2*, *Pou3f3*, *Mdga1, Mef2c and Cux1*) in uncommitted E11.5 cells (grey), or in cells committed to the deep- (cyan; red line) or upper-layer trajectory (green; blue line). The number of cells analyzed in (**c** and **d**) can be deduced from the clusters listed in Supplementary table 3. Violin plots are inset by a dot at the group mean and a vertical line showing standard error. P-values, top to bottom in (**c**), 7.27e-4, n.s. and 0.03 and in (**d**) 0.013, 0.029 and 1.47e-4. All statistics are based on two-tailed t-tests, stars indicate significant differences between indicated groups, with * = $p<0.05$, ** = $p<0.01$ and *** = $p<0.001$.

Supplementary Fig. 4 (Hagey et al).

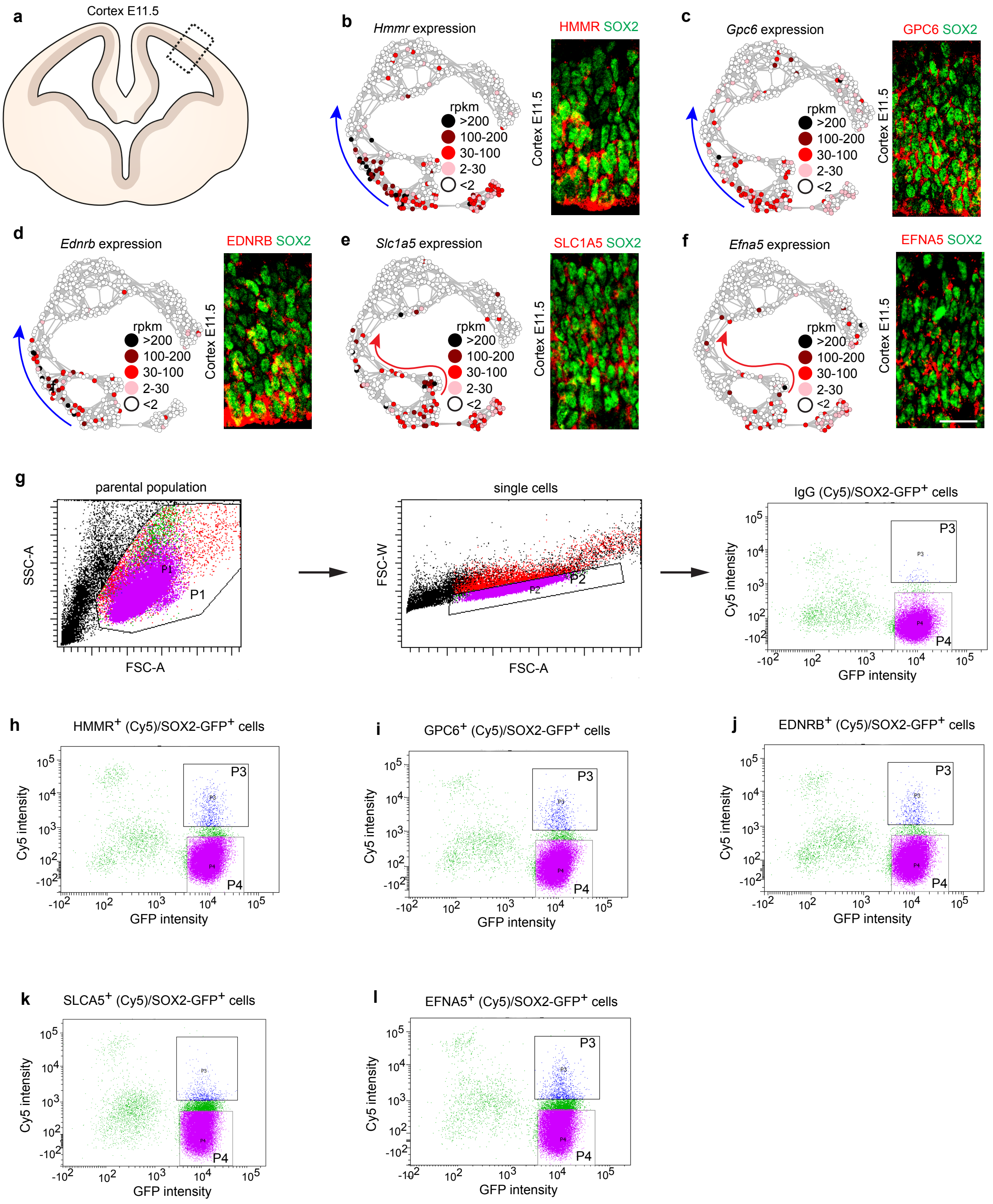

Supplementary Fig. 4 FACS based isolation of cortical progenitor populations. **a-f** *t*SNE-NN maps coloured by *Hmmr* (**b**), *Gpc6* (**c**), *Ednrb* (**d**), *Slc1a5* (**e**) or *Efna5* (**f**) expression levels. Immunohistochemistry show expression of SOX2 (green) together with HMMR (**b**), GPC6 (**c**), EDNRB (**d**), SLC1A5 (**e**) or EFNA5 (**f**) (red) in the E11.5 cortex (**a**). Immunohistochemistry shown in (**b-f**) was repeated 3 times. **g-l** FACS plots showing isolation efficiency of *Sox2*-GFP^{+} cells labelled with IgG control (**g**), HMMR (**h**), GPC6 (**i**), EDNRB (**j**), SLC1A5 (**k**) or EFNA5 (**l**) antibodies. Scale bar represents 15 µm in (**b-f**).

Supplementary Fig. 5 (Hagey et al).

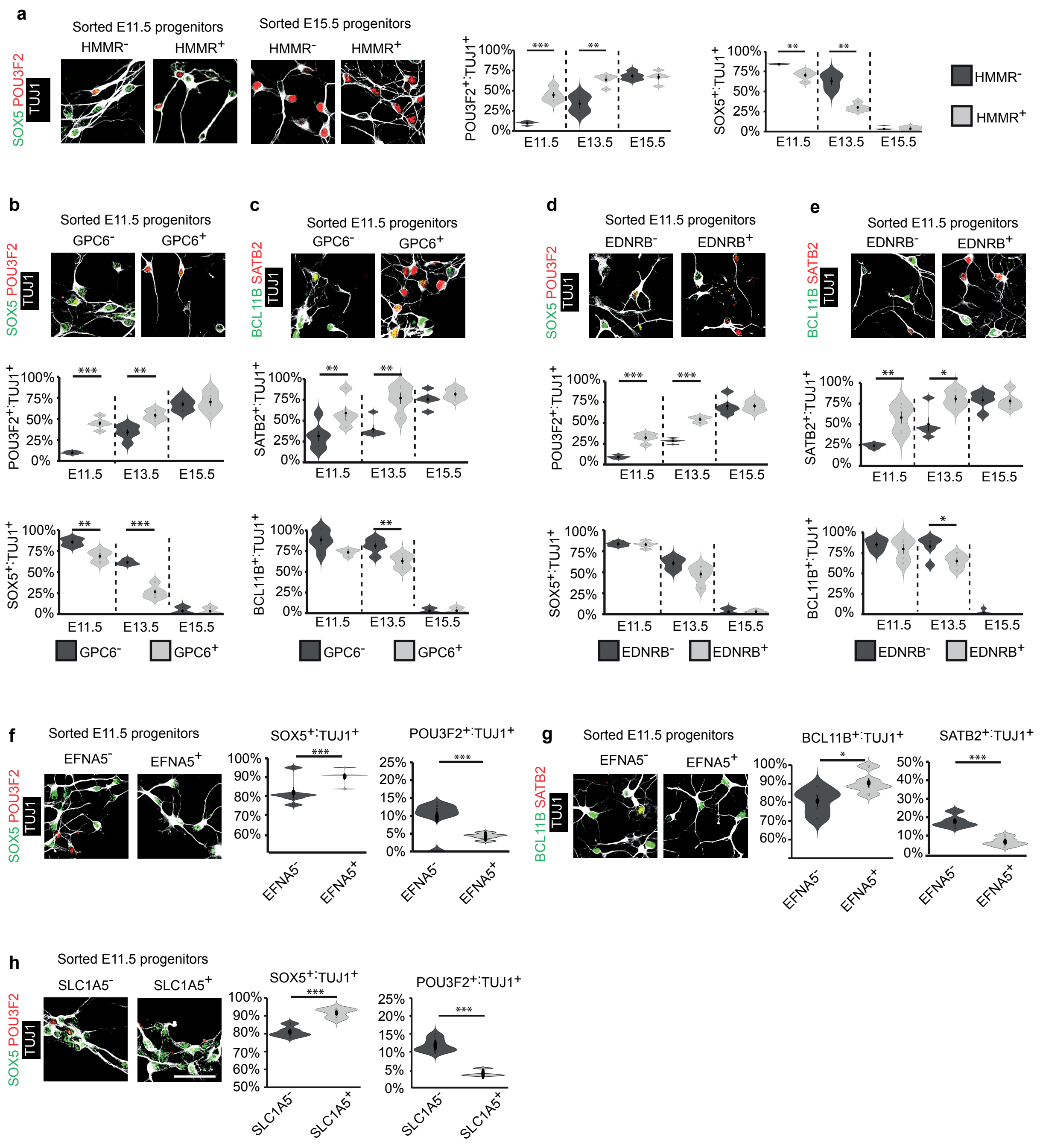

Supplementary Fig. 5 Characterization of uncommitted and committed cortical progenitors. **a-e** SOX5 (green) and POU3F2 (red) or BCL11B (green) and SATB2 (red) expression in TUJ1^{+} (white) neurons derived from E11.5 (n=5 biological independent experiments), E13.5 (n=4 biological independent experiments) and E15.5 (n=5 biological independent experiments) HMMR^{-} and HMMR^{+} (**a**), GPC6^{-} and GPC6^{+} (n=5 biological independent experiments; **b, c**) or EDNRB^{-} and EDNRB^{+} (n=5 biological independent experiments; **d, e**) cortical progenitors after two days of *in vitro* differentiation. P-values in (**a**), differences in POU3F2^{+} neurons at E11.5 4.27e-4, E13.5 4.81e-3 and in SOX5^{+} neurons at E11.5 5.46e-3, E13.5 2.83e-3. P-values in (**b**), differences in POU3F2^{+} neurons at E11.5 1.89e-4, E13.5 3.5e-3 and in SOX5^{+} neurons at E11.5 2.08-3, E13.5 1.56e-4. P-values in (**c**), differences in SATB2^{+} neurons at E11.5 2.61e-3, E13.5 3.05e-3 and in BCL11B^{+} neurons at E13.5 9.12e-3. P-values in (**d**), differences in POU3F2^{+} neurons at E11.5 2.72-4, E13.5 1.76e-6. P-values in (**e**), differences in SATB2^{+} neurons at E11.5 8.92e-3, E13.5 0.016 and in BCL11B^{+} neurons at E13.5 0.041. **f, g** SOX5 (green) and POU3F2 (red) or BCL11B (green) and SATB2 (red) expression in TUJ1^{+} (white) neurons derived from isolated E11.5 EFNA5^{-} and EFNA5^{+} cortical progenitors after two days of *in vitro* differentiation (n=5 biological independent experiments). P-values in (**f**), differences in SOX5^{+} neurons 2.91e-4 and in POU3F2^{+} neurons 2.02e-5. P-values in (**g**), differences in BCL11B^{+} neurons 0.04 and in SATB2^{+} neurons 5.49e-4. **h** SOX5 (green) and POU3F2 (red) expression in TUJ1^{+} (white) neurons derived from isolated E11.5 SLC1A5^{-} and SLC1A5^{+} cortical progenitors after two days of *in vitro* differentiation (n=5 biological independent experiments). P-values in SOX5^{+} neurons 3.21e-4 and in POU3F2^{+} neurons 1.46e-4. Scale bar represents 20 μm in (**b-e** and **f-h**). Violin plots are inset by rings corresponding to the individual data points, a filled dot at the group mean and a vertical line showing standard error. All statistics are based on two-tailed t-tests, stars indicate significant differences between indicated

groups, with * = $p<0.05$, ** = $p<0.01$, and *** = $p<0.001$. Source data are provided as a Source Data file.

Supplementary Fig. 6 (Hagey et al).

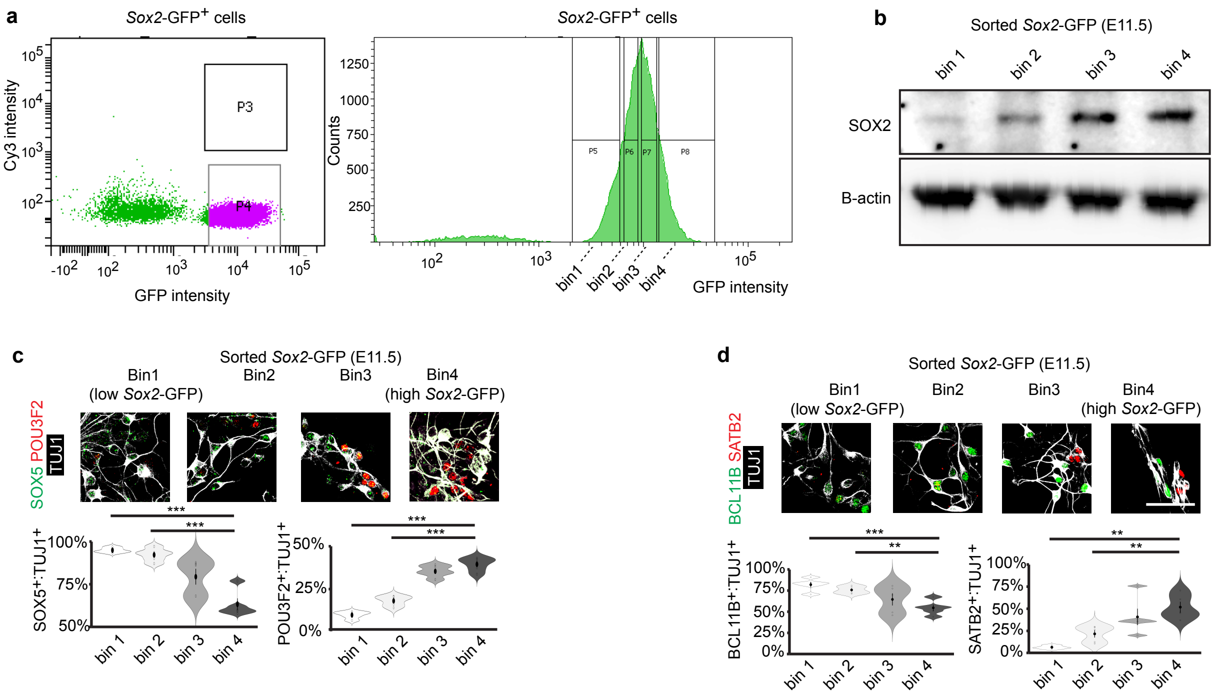

Supplementary Fig. 6 SOX2 expression levels distinguishes cortical progenitor populations. **a** E11.5 cortical cells were separated into four bins based on their expression level of *Sox2*-GFP using FACS. Bin 1 represents cells expressing low levels of *Sox2*-GFP and bin 4 cells expressing the highest levels of *Sox2*-GFP. **b** Immunoblot analysis of SOX2 expression levels in E11.5 cortical progenitors separated based on their levels of *Sox2*-GFP. N= 3 biological independent experiments. **c, d** Immunohistochemistry and quantifications of SOX5 (green), BCL11B (green), POU3F2 (red) and SATB2 (red) in $TUJ1^+$ (white) neurons derived from E11.5 *Sox2*-GFP progenitors (Bin 1 to 4), after two days of *in vitro* differentiation (n=5 biological independent experiments). P-values for differences in generated $SOX5^+$ neurons (**c**) between bin 1 and bin 4 cells is 6.14e-4, between bin 2 and bin4 cells 3.45e-4. P-values for differences in generated $POU3F2^+$ neurons (**c**) between bin 1 and bin 4 cells is 4.82e-6, between bin 2 and bin 4 cells 1.7-5. P-values for differences in generated $BCL11B^+$ neurons (**d**) between bin 1 and bin 4 cells is 6.8e-4, between bin2 and bin 4 cells 2.8e-3. P-values for differences in generated $SATB2^+$ neurons (**d**) between bin 1 and bin 4 cells is 1.51e-3, between bin 2 and bin 4 cells 4.39e-3. Scale bar represents 20 µm in (**c** and **d**). Violin plots are inset by rings corresponding to the individual data points, a filled dot at the group mean and a vertical line showing standard error. All statistics are based on two-tailed t-tests, stars indicate significant differences between indicated groups, with ** = $p<0.01$ and *** = $p<0.001$. Source data are provided as a Source Data file.

Supplementary Fig. 7 (Hagey et al).

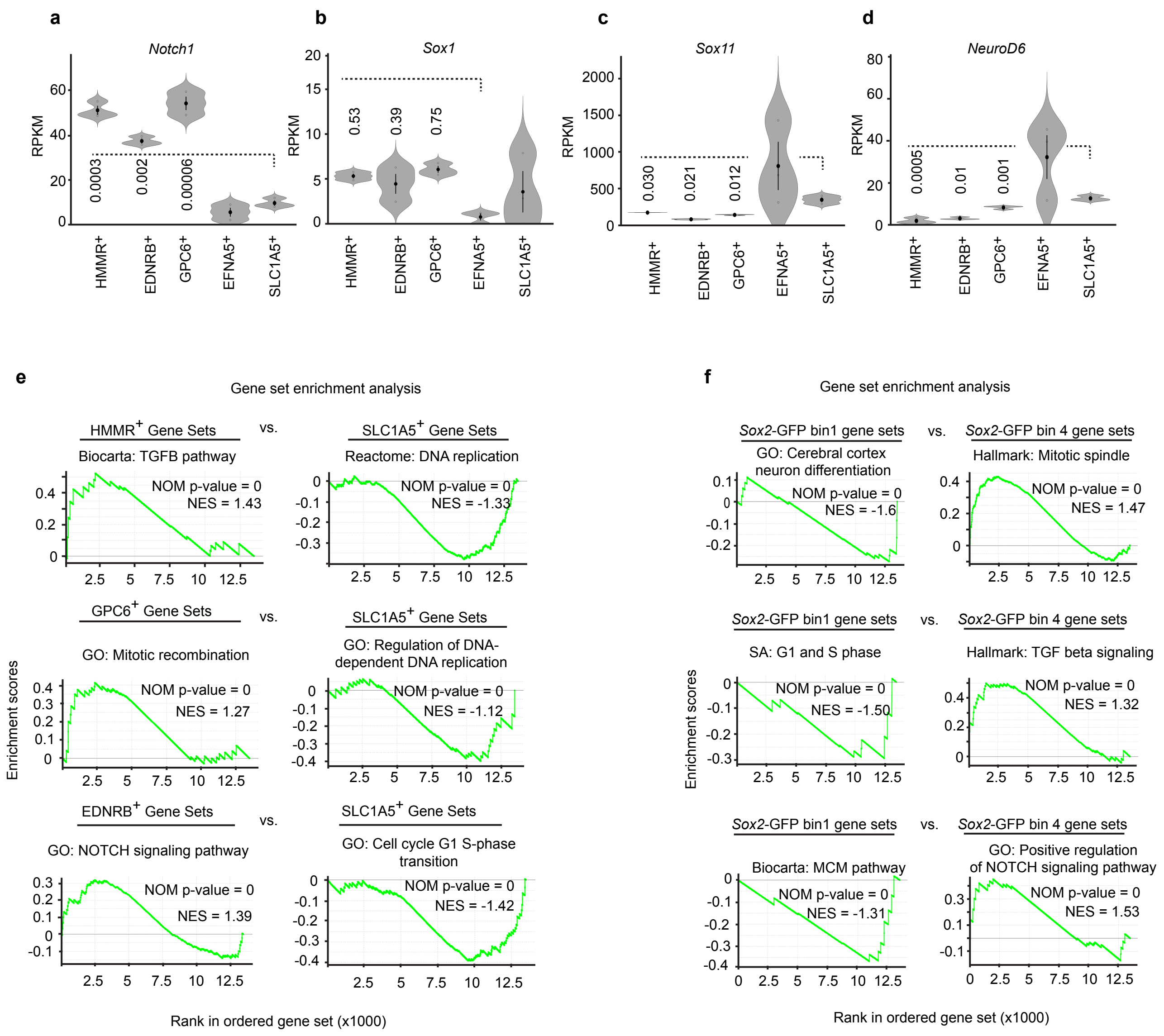

Supplementary Fig. 7 Extended bioinformatic confirmation of sorted progenitor characteristics. **a-d** Violin plots of RPKMs for the RGC associated genes *Notch1* (**a**) and *Sox1* (**b**), and the differentiation genes *Sox11* (**c**) and *NeuroD6* (**d**). N=3 biological independent experiments. Violin plots are inset by rings corresponding to the individual data points, a filled dot at the group mean and a vertical line showing standard error. P-values displayed are based on two-tailed t-tests between the groups indicated. **e, f** GSEA-derived GO-terms based on RNA-sequencing of sorted HMMR$^+$, GPC6$^+$, EDNRB$^+$ versus SLC1A5$^+$ E11.5 cortical cells (**e**), or of sorted E11.5 cortical cells expressing low levels of *Sox2*-GFP (bin 1) versus cells expressing high levels of *Sox2*-GFP (bin 4) (**f**). **N**ominal (NOM) p-values and normalized enrichment scores (NES) are indicated.

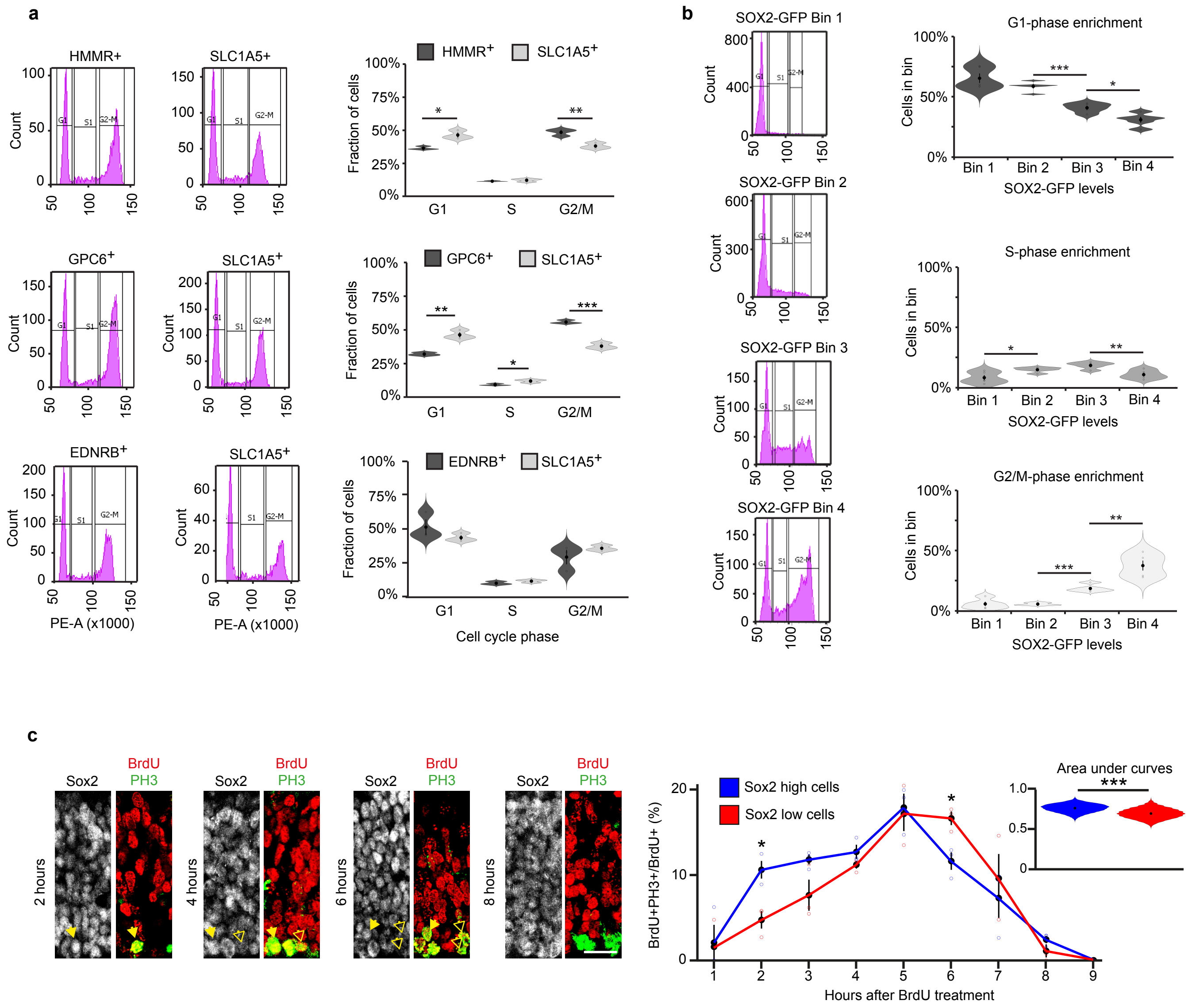
a
HMMR+
SLC1A5+
GPC6+
EDNRB+
Count
PE-A (x1000)
G1
S1
G2-M
Fraction of cells
Cell cycle phase
G2/M
b
SOX2-GFP Bin 1
SOX2-GFP Bin 2
SOX2-GFP Bin 3
SOX2-GFP Bin 4
G1-phase enrichment
S-phase enrichment
G2/M-phase enrichment
Cells in bin
Bin 1
Bin 2
Bin 3
Bin 4
SOX2-GFP levels
c
Sox2
BrdU
PH3
2 hours
4 hours
6 hours
8 hours
Sox2 high cells
Sox2 low cells
BrdU+PH3+/BrdU+ (%)
Hours after BrdU treatment
Area under curves

Supplementary Fig. 8 RGCs are enriched within the G2/M phase of the cell-cycle. **a, b** FACS analysis and quantification of cell-cycle phase enrichment by propidium iodide treatment of sorted HMMR$^+$, GPC6$^+$, EDNRB$^+$ and SLC1A5$^+$ E11.5 cortical cells (**a**) or *Sox2*-GFP bin 1-4 E11.5 cortical cells. P-values (**a**); HMMR$^+$ vs. SLC1A5$^+$ cells, fraction in G1 0.021 and fraction in G2/M 6.33e-3. P-values (**a**); GPC6$^+$ vs. SLC1A5$^+$ cells, fraction in G1 9.33e-3, fraction in S 0.036 and fraction in G2/M 9.08e-4. P-values (**b**); G1-phase enrichment of SOX2-GFP$^+$ cells, bin 2 vs. bin 3 cells is 9.85e-5 and between bin 3 vs. bin 4 cells 0.012. P-values (**b**); S-phase enrichment of SOX2-GFP$^+$ cells, bin 1 vs. bin 2 cells 0.033, between bin 3 vs. bin 4 cells 6.29e-3. P-values (**b**); G2/M-phase enrichment of SOX2-GFP$^+$ cells, bin 2 vs. bin 3 cells 2.82-4, between bin 3 vs. bin 4 cells 7.61e-3. N=8797 number HMMR$^+$ cells, 11309 GPC6$^+$ cells, 3149 EDNRB$^+$ cells and 6218 SLC1A5$^+$ cells over 3 independent experiments or N=15575 of *Sox2*-GFP$^+$ bin 1 cells, 17973 bin 2 cells, 11278 bin 3 cells and 13448 bin 4 cells over 5 independent experiments. Violin plots are inset by rings corresponding to the individual data points, a filled dot at the group mean and a vertical line showing standard error. Statistics (stars) show differences in cell-cycle phase distribution between sorted populations (**a**) or starred group and that of the next highest bin (**b**). **c** Immunohistochemistry and quantification of SOX2 (white), PH3 (green) and BrdU (red) at various time points following BrdU injection. Inset violin plot shows the average area under SOX2 high (blue) and low (red) cell PH3/BrdU curves, Hausdorff Distance = 1.83. Immunohistochemistry was repeated 3 times. Stars indicate significant differences between SOX2 high and low cells at time point indicated. P-values in (**c**), left to right, 0.016 and 0.02. Scale bar represents 15 μm in (**c**). All statistics are based on two-tailed t-tests with * = $p<0.05$, ** = $p<0.01$, and *** = $p<0.001$. Source data are provided as a Source Data file.

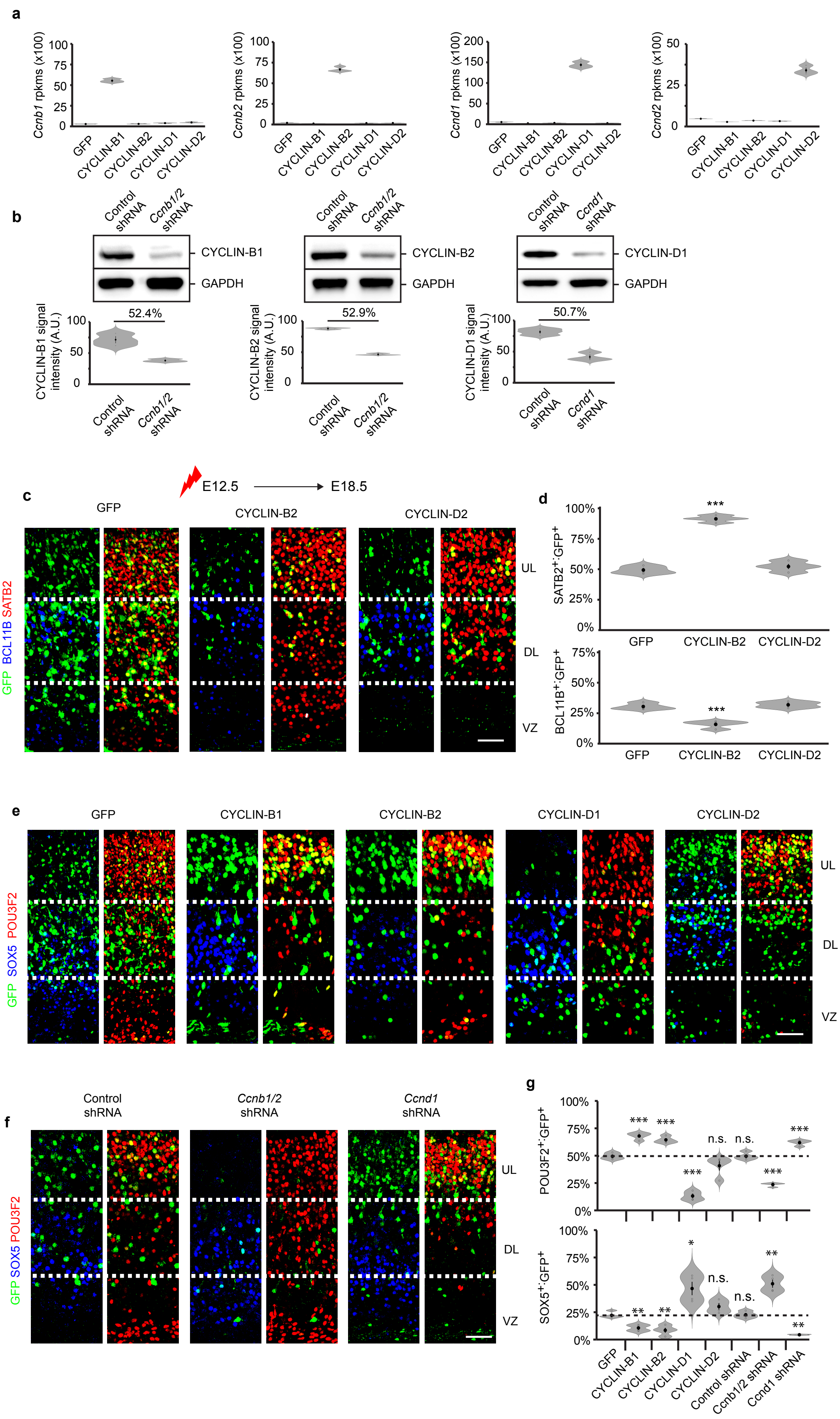

a
Ccnb1 rpkms (x100)
Ccnb2 rpkms (x100)
Ccnd1 rpkms (x100)
Ccnd2 rpkms (x100)
GFP
CYCLIN-B1
CYCLIN-B2
CYCLIN-D1
CYCLIN-D2
b
Control shRNA
Ccnb1/2 shRNA
Ccnd1 shRNA
CYCLIN-B1
CYCLIN-B2
CYCLIN-D1
GAPDH
52.4%
52.9%
50.7%
CYCLIN-B1 signal intensity (A.U.)
CYCLIN-B2 signal intensity (A.U.)
CYCLIN-D1 signal intensity (A.U.)
c
E12.5
E18.5
GFP BCL11B SATB2
UL
DL
VZ
d
SATB2+:GFP+
BCL11B+:GFP+
***
e
GFP SOX5 POU3F2
f
g
POU3F2+:GFP+
SOX5+:GFP+
n.s.
Control shRNA
Ccnb1/2 shRNA
Ccnd1 shRNA

Supplementary Fig. 9 CYCLIN-B1/2 and -D1 promote E12.5 progenitors to commit to upper- and deep-layer neurogenesis, respectively. **a** Violin plots of *Ccnb1*, *Ccnb2*, *Ccnd1* and *Ccnd2* RPKMs in sorted E13.5 cortical cells electroporated with CYCLIN-B1, CYCLIN-B2, CYCLIN-D1, CYCLIN-D2 or GFP expressing constructs at E12.5. N=3 biological independent experiments. **b** Immunoblotting for CYCLIN-B1, CYCLIN-B2 or CYCLIN-D1 24 hours after transfection with Control shRNA*, Ccnb1/2* shRN*A* or *Ccnd1* shRNA in P19 cells. Violin plots show quantification of CYCLIN-B1, CYCLIN-B2 and CYCLIN-D1 knock-down based on band intensities of immunoblots from triplicate experiments. **c, d** Immunohistochemistry (**c**) and quantifications (**d**) of BCL11B (blue) and SATB2 (red) in E18.5 cortices electroporated with CYCLIN-B2, CYCLIN-D2 or GFP (green) at E12.5. Quantifications show the percentage of GFP^+ cells expressing BCL11B or SATB2 (n=4 biological independent experiments). P-values, CYCLIN-B2 electroporation vs. GFP; BCL11B 3.05e-4, SATB2 9.67e-7. N=3 biologically independent experiments. **e, f, g** Immunohistochemistry (**e, f**) and quantifications (**g**) of SOX5 (blue) and POU3F2 (red) in E18.5 cortices electroporated with CYCLIN-B1, CYCLIN-B2, CYCLIN-D1, CYCLIN-D2, control shRNA*, Ccnb1/2* shRNA, *Ccnd1* shRNA or GFP (green) at E12.5. Quantifications show the percentage of GFP^+ cells expressing SOX5 or POU3F2 (n=4 biological independent experiments). P-values, SOX5 expression (vs. GFP); CYCLIN-B1 1.37e-3, CYCLIN-B2 3.33e-3, CYCLIN-D1 0.025, *Ccnb1/2* shRNA 1.94e-3, *Ccnd1* shRNA 1.01e-3; SATB2 9.67e-7. P-values, POU3F2 expression (vs. GFP); CYCLIN-B1 1.28e-4, CYCLIN-B2 3.23e-4, CYCLIN-D1 1.43e-5, *Ccnb1/2* shRNA 1.54e-5, *Ccnd1* shRNA 5.43e-4. N=3 biologically independent experiments. Scale bar represents 40 µm in (**c, e** and **f**). Violin plots are inset by rings corresponding to the individual data points, a filled dot at the group mean and a vertical line showing standard error. Stars indicate significant differences between indicated group and GFP based on two-tailed t-tests, with * = p<0.05, ** = p<0.01 and *** = p<0.001. Source data are provided as a Source Data file.

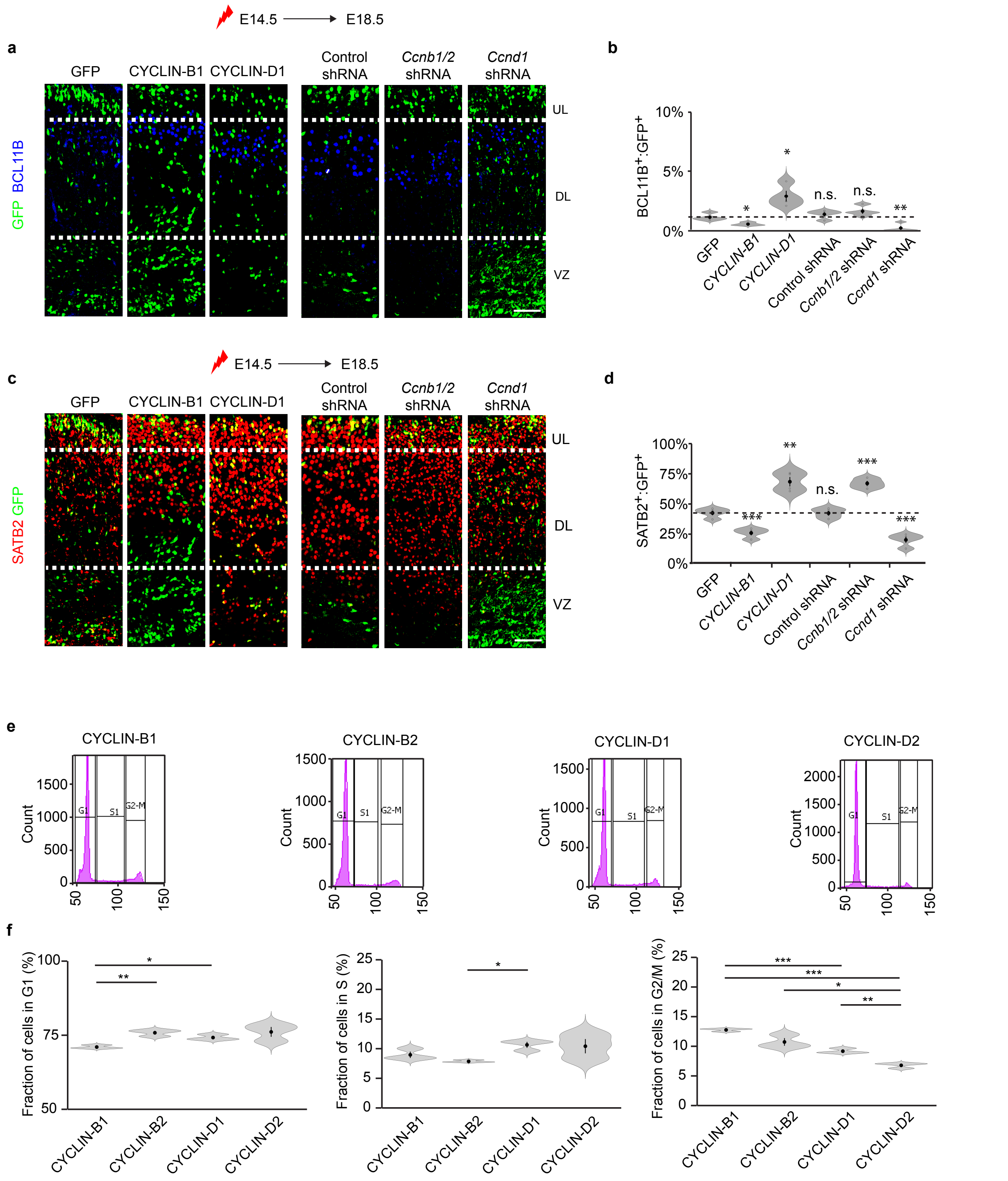

E14.5 → E18.5
a
GFP
CYCLIN-B1
CYCLIN-D1
Control shRNA
Ccnb1/2 shRNA
Ccnd1 shRNA
GFP BCL11B
UL
DL
VZ
b
BCL11B+:GFP+
10%
5%
0%
*
*
n.s.
n.s.
**
GFP
CYCLIN-B1
CYCLIN-D1
Control shRNA
Ccnb1/2 shRNA
Ccnd1 shRNA
c
SATB2 GFP
d
SATB2+:GFP+
100%
75%
50%
25%
0%
**
***
n.s.
***
***
e
CYCLIN-B1
CYCLIN-B2
CYCLIN-D1
CYCLIN-D2
Count
G1
S1
G2-M
50
100
150
f
Fraction of cells in G1 (%)
Fraction of cells in S (%)
Fraction of cells in G2/M (%)

Supplementary Fig. 10 CYCLIN-B1/2 delay and -D1 promotes cortical neurogenesis. **a, b** Immunohistochemistry (**a**) and quantifications (**b**) of BCL11B (blue) in E18.5 cortices electroporated with vectors expressing GFP (green) either alone or together with CYCLIN-B1, CYCLIN-D1, control shRNA*, Ccnb1/2* shRNA or *Ccnd1* shRNA at E14.5. Quantifications show the percentage of GFP^+ cells labelled with BCL11B. P-values, BCL11B expression (vs. GFP); CYCLIN-B1 0.023, CYCLIN-D1 0.023, *Ccnd1* shRNA 7.31e-3. N=4 biological independent experiments. **c, d** Immunohistochemistry (**c**) and quantifications (**d**) of SATB2 (red) in E18.5 cortices electroporated with vectors expressing GFP (green) either alone or together with CYCLIN-B1, CYCLIN-D1, control shRNA*, Ccnb1/2* shRNA or *Ccnd1* shRNA at E14.5. Quantifications show the percentage of GFP^+ cells labelled with SATB2. P-values, SATB2 expression (vs. GFP); CYCLIN-B1 9.8e-4, CYCLIN-D1 2.98e-3, *Ccnb1/2* shRNA 1.13e-4, *Ccnd1* shRNA 5.86e-4. N=4 biological independent experiments. **e, f** FACS analysis (**e**) and quantification (**f**) of cell-cycle phase enrichment by propidium iodide treatment of sorted E13.5 cortical cells 20 hrs after electroporation with CYCLIN-B1, CYCLIN-B2, CYCLIN-D1 or CYCLIN-D2. N=40027 of CYCLIN-B1 electroporated cells, 32516 CYCLIN-B2 electroporated cells, 31853 CYCLIN-D1 electroporated cells, 31326 CYCLIN-D2 electroporated cells and 33353 GFP electroporated cells over 3 independent experiments. P-values; G1-phase enrichment of electroporated cells, CYCLIN-B1 vs. CYCLIN-B2 9.85e-5, CYCLIN-B1 vs. CYCLIN-D1 0.012. P-values; S-phase enrichment of electroporated cells, CYCLIN-B2 vs. CYCLIN-D1 0,026. P-values; G2/M-phase enrichment of electroporated cells, CYCLIN-B1 vs. CYCLIN-D1 6.73e-4, CYCLIN-B1 vs. CYCLIN-D2 1.84e-4, CYCLIN-B2 vs. CYCLIN-D2 0.015, CYCLIN-D1 vs. CYCLIN-D2 2.57e-3. Scale bar represents 40 µm in (**a** and **c**). Violin plots are inset by rings corresponding to the individual data points, a filled dot at the group mean and a vertical line showing standard error. Stars indicate significant differences between indicated group and GFP (**b, d**) or indicated groups (**f**) based on two-tailed

t-tests, with * = p<0.05, ** = p<0.01 and *** = p<0.001. Source data are provided as a Source Data file.

Supplementary Fig. 11 (Hagey et al).

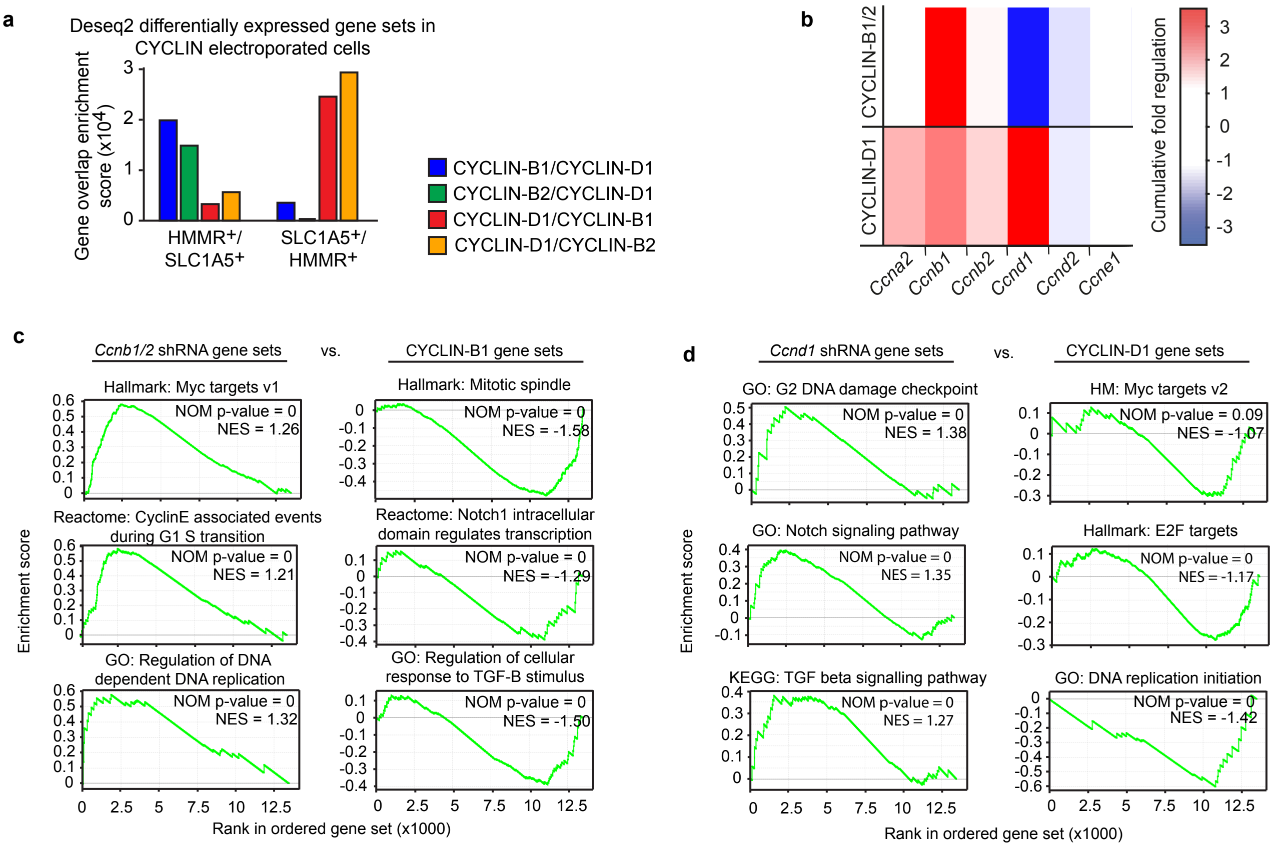

Supplementary Fig. 11 Extended bioinformatic analysis of CYCLIN-B1/2 and -D1 modulation. **a** Gene overlap enrichment analysis of genes upregulated in RNA-sequencing of CYCLIN-B1 over CYCLIN-D1 (blue bars), CYCLIN-B2 over CYCLIN-D1 (green bars), CYCLIN-D1 over CYCLIN-B1 (red bars) or CYCLIN-D1 over CYCLIN-B2 (orange bars) electroporated cells and genes overrepresented in $HMMR^+$ over $SLC1A5^+$ (left chart) or $SLC1A5^+$ over $HMMR^+$ (right chart) sorted cell populations, as determined by Deseq2. **b** Cumulative effect on cyclin gene expression 24 hrs after overexpression of CYCLIN-B1/2 or CYCLIN-D1, and knockdown of *Ccnb1/2* or *Ccnd1* in E12.5 cortices. **c** GSEA-derived GO-terms based on RNA-sequencing of cortical cells electroporated with *Ccnb1/2* shRNA compared with those electroporated with CYCLIN-B1. **d** GSEA-derived GO-terms based on RNA-sequencing of cortical cells electroporated with *Ccnd1* shRNA compared with those electroporated with CYCLIN-D1.

Supplementary Fig. 12 (Hagey et al).

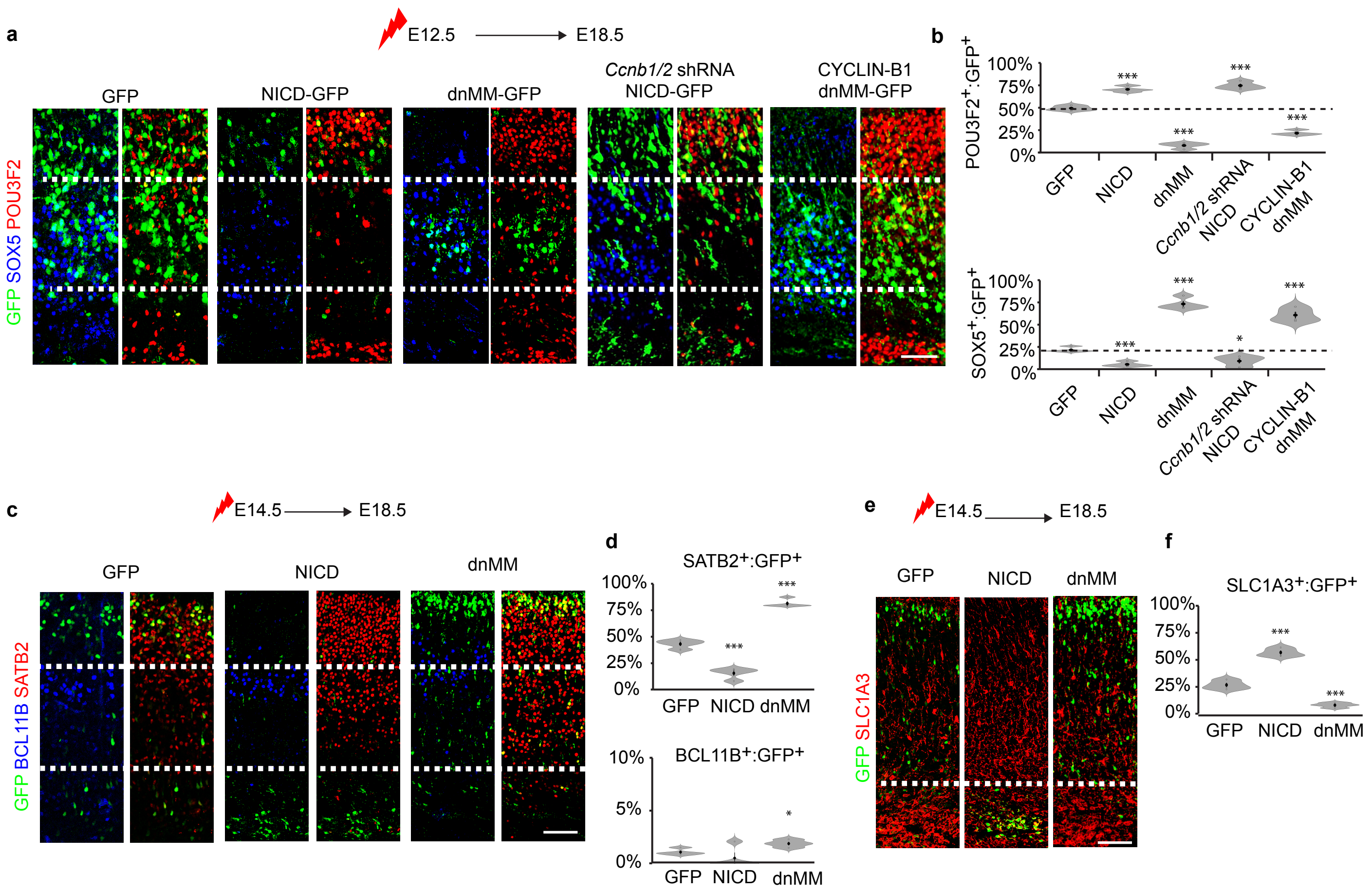

Supplementary Fig. 12 CYCLIN-B1/2 delay cortical lineage commitment via the NOTCH pathway. **a, b** Immunohistochemistry (**a**) and quantifications (**b**) of SOX5 (blue) and POU3F2 (red) in E18.5 cortices after misexpressing GFP, NICD with or without *Ccnb1/2* shRNA, or dnMM with or without CYCLIN-B1 (green; n=4 biological independent experiments) at E12.5. P-values, SOX5 expression (vs. GFP); NICD 1.84e-4, dnMM 9.96e-5, *Ccnb1/2* shRNA/NICD 0.012, CYCLIN-B1/dnMM 6.78e-4. P-values, POU3F2 expression (vs. GFP); NICD 5.67e-5, dnMM 1.29e-6, *Ccnb1/2* shRNA/NICD 9.82e-5, CYCLIN-B1/dnMM 7.05e-6. **c, d** Immunohistochemistry (**c**) and quantifications (**d**) of BCL11B (blue) and SATB2 (red) in E18.5 cortices electroporated with GFP, NICD or dnMM (green; n=4 biological independent experiments) at E14.5. P-values, BCL11B expression (vs. GFP); dnMM 0.012. P-values, SATB2 expression (vs. GFP); NICD 1.93e-4, dnMM 9.2e-6. **e, f** Immunohistochemistry (**e**) and quantifications (**f**) of SLC1A3 in E18.5 cortices electroporated with GFP, NICD or dnMM (green; n=4 biological independent experiments) at E14.5. P-values, SLC1A3 expression (vs. GFP); NICD 3.21e-5 and dnMM 6.03e-4. Scale bar represents 35 μm in (**a**) and 70 μm in (**c** and **e**). Violin plots are inset by rings corresponding to the individual data points, a filled dot at the group mean and a vertical line showing standard error. Stars indicate significant differences between indicated group and GFP based on two-tailed t-tests, with * = $p<0.05$ and *** = $p<0.001$. Source data are provided as a Source Data file.

Uncropped original scans

Supplementary Fig. 6b

a

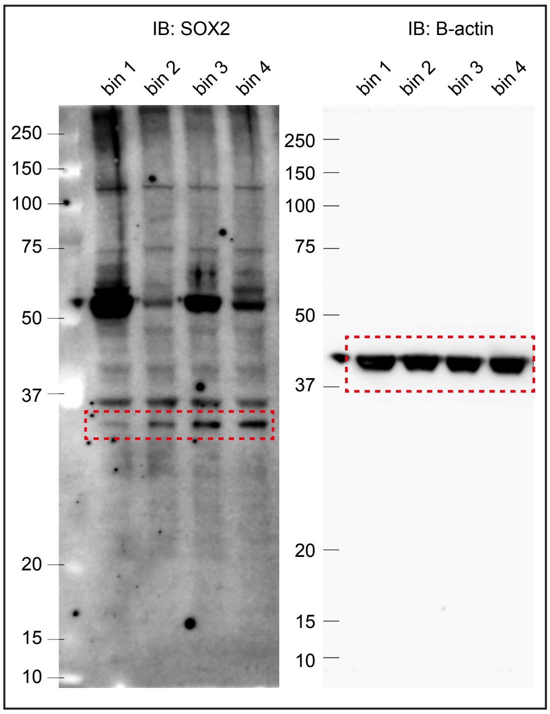

Supplementary Fig. 9b

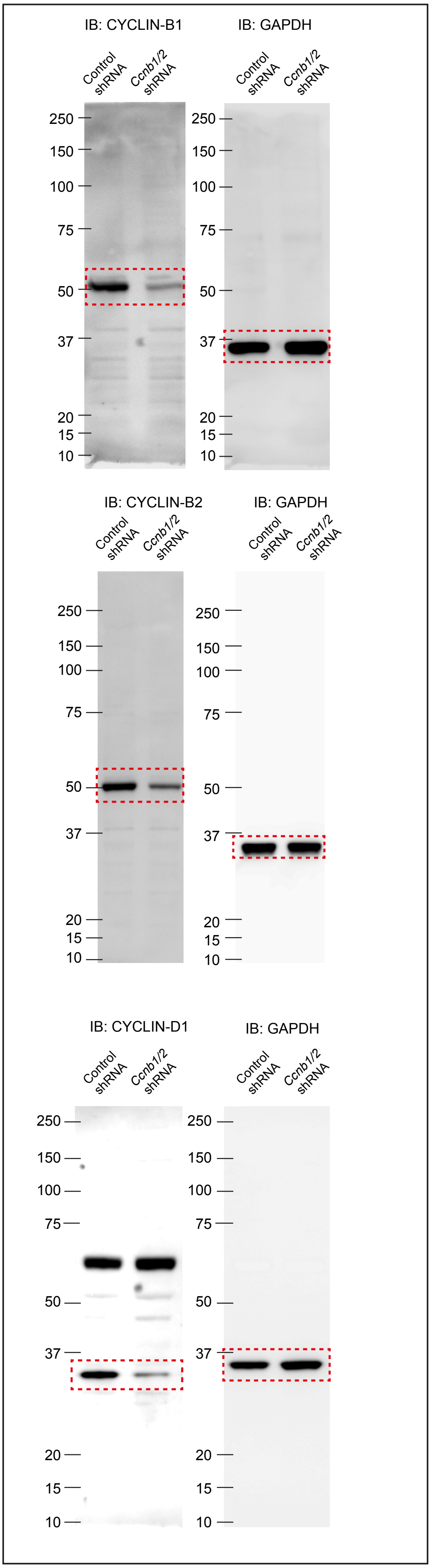

Supplement: Supplementary file 1 — Supplementary Information [file 41467_2020_16597_MOESM1_ESM.pdf]
