# Supplementary material for: CYCLIN-B1/2 and -D1 act in opposition to coordinate cortical progenitor self-renewal and lineage commitment
Source: Nat Commun. 2020 Jun 9;11:2898. doi: 10.1038/s41467-020-16597-8 (PMC7283355; doi:10.1038/s41467-020-16597-8)
Supplement: Supplementary file 3 — Reporting Summary [file 41467_2020_16597_MOESM3_ESM.pdf]

nature research

Corresponding author(s): Daniel W. Hagey and Jonas Muhr

Last updated by author(s): Apr 28, 2020

Reporting Summary

Nature Research wishes to improve the reproducibility of the work that we publish. This form provides structure for consistency and transparency in reporting. For further information on Nature Research policies, see Authors & Referees and the Editorial Policy Checklist.

Statistics

For all statistical analyses, confirm that the following items are present in the figure legend, table legend, main text, or Methods section.

n/a	Confirmed	
☐	☒	The exact sample size (*n*) for each experimental group/condition, given as a discrete number and unit of measurement
☐	☒	A statement on whether measurements were taken from distinct samples or whether the same sample was measured repeatedly
☐	☒	The statistical test(s) used AND whether they are one- or two-sided<br>*Only common tests should be described solely by name; describe more complex techniques in the Methods section.*
☒	☐	A description of all covariates tested
☒	☐	A description of any assumptions or corrections, such as tests of normality and adjustment for multiple comparisons
☐	☒	A full description of the statistical parameters including central tendency (e.g. means) or other basic estimates (e.g. regression coefficient) AND variation (e.g. standard deviation) or associated estimates of uncertainty (e.g. confidence intervals)
☐	☒	For null hypothesis testing, the test statistic (e.g. *F*, *t*, *r*) with confidence intervals, effect sizes, degrees of freedom and *P* value noted<br>*Give P values as exact values whenever suitable.*
☒	☐	For Bayesian analysis, information on the choice of priors and Markov chain Monte Carlo settings
☒	☐	For hierarchical and complex designs, identification of the appropriate level for tests and full reporting of outcomes
☒	☐	Estimates of effect sizes (e.g. Cohen's *d*, Pearson's *r*), indicating how they were calculated

Our web collection on statistics for biologists contains articles on many of the points above.

Software and code

Policy information about availability of computer code

Data collection	Mapping was performed using Star v2.5 and RPKMs were calculated using rpkmforgenes.py (Ramskold et al. 2009). Cell filtering was performed using custom code to remove cells with <200000 mapped reads, 1500 < detected genes > 9000 or an average cell-cell correlation (R package hclust command cor) coefficient >3 s<br><br>d from the mean.
Data analysis	R (3.5.3) package Rtsne (0.15) was used to create weighted PCA scores from which the Euclidean distance between each cell's ten nearest neighbors was used to construct an adjacency matrix with edges weighted according to [(Graph max Euclidean distance – pairwise Euclidian distance) / Graph max Euclidian distance]. The directed weighted adjacency matrix was then visualized using the R package Igraph (1.4.2.1) to create a force directed graph network of all cells in the data set. R package SCDE (2.14.0) was used to extract differentially expressed genes from Igraph infomap clusters. Genes with a Stouffer's Z-score above 0.4 (from https://en.wikipedia.org/wiki/Fisher's_method) were then used as input into the R package WGCNA (1.67) to form clusters of similarly expressed genes. R package prcomp was used to perform PCA on bulk RNA-seq data sets and cortical cells for maturation stage assignment. R package Deseq2 (1.26) was used to calculate genes differentially expressed between bulk RNA-seq data sets. Sorted cell population and electroporation experiments were compared as indicated using GSEA software from [http://software.broadinstitute.org/]. Pantherdb.org GO biological functions complete were used for gene ontology analysis. GSEA (http://software.broadinstitute.org/) was used to find GO terms overrepresented between bulk RNA-seq data sets. Pseudotime (DDRTree) were performed using Monocle 2.14.0, with cell pseudotime ranks correlated to those from our maturation stage assignments. Level of SOX2 expression was determined using ImageJ (1.52).

For manuscripts utilizing custom algorithms or software that are central to the research but not yet described in published literature, software must be made available to editors/reviewers. We strongly encourage code deposition in a community repository (e.g. GitHub). See the Nature Research guidelines for submitting code & software for further information.

Data

Policy information about availability of data

All manuscripts must include a data availability statement. This statement should provide the following information, where applicable:

- Accession codes, unique identifiers, or web links for publicly available datasets
- A list of figures that have associated raw data
- A description of any restrictions on data availability

All data used in this work is available under NCBI accession SRP132833 [https://www.ncbi.nlm.nih.gov/sra/?term=SRP132833]. All data supporting the findings and custom code within this paper are available from the corresponding authors upon reasonable request. The source data underlying Figs. 3-9 and Supplementary Figs. 5, 6, 8-10 and 12 are provided as a Source Data file.

Field-specific reporting

Please select the one below that is the best fit for your research. If you are not sure, read the appropriate sections before making your selection.

☒ Life sciences ☐ Behavioural & social sciences ☐ Ecological, evolutionary & environmental sciences

For a reference copy of the document with all sections, see nature.com/documents/nr-reporting-summary-flat.pdf

Life sciences study design

All studies must disclose on these points even when the disclosure is negative.

Sample size	No sample size calculation was performed and was standardised for common experiments. Our experience with the experiments applied in this work has demonstrated to us that significant results are clear after three independent repetitions. Thus, we performed one to two additional experiments above this to be certain of their consistency and reproducibility.
Data exclusions	No data were excluded
Replication	All attempts at data replication were successful. All n's in this work represent independent experiments. The consistency of these results is thus represented in the statistically significant differences we find between experimental groups.
Randomization	As all experimental subjects were from the inbred CD1 or C57 mouse lines, assignment of treatments was random within this limited background.
Blinding	No blinding protocol was performed, as no a priori knowledge was assumed about the groupsanalysed. Key experiments were performed and analysed independently by different researchers, who arrived at the same results. This gave us confidence to proceed with our analysis under the standardised experimental framework agreed upon.

Reporting for specific materials, systems and methods

We require information from authors about some types of materials, experimental systems and methods used in many studies. Here, indicate whether each material, system or method listed is relevant to your study. If you are not sure if a list item applies to your research, read the appropriate section before selecting a response.

Materials & experimental systems

n/a	Involved in the study
☐	☒ Antibodies
☐	☒ Eukaryotic cell lines
☒	☐ Palaeontology
☐	☒ Animals and other organisms
☒	☐ Human research participants
☒	☐ Clinical data

Methods

n/a	Involved in the study
☒	☐ ChIP-seq
☐	☒ Flow cytometry
☒	☐ MRI-based neuroimaging

Antibodies

Antibodies used	SOX2 (sc-17320, Santa Cruz), Phospho-Histone H3 (clone 3H10, Millipore), EOMES (ab23345, Abcam), BCL11B (ab18465, Abcam), SATB2 (ab92446, Abcam), SOX5 (Generated by "The Ludwig Institute for Cancer Research", Muhr laboratory), POU3F2 (sc-6029, Santa Cruz), TUJ1 (ab41489, Abcam), GPC6 (bs-2177R-A647, Bioss), HMMR (bs-4736R-A647, Bioss), EDNRB (bs-2363R-A647, Bioss), EFNA5 (bs-6048R-A647, Bioss) and SLC1A5 (bs-0473-A647, Bioss), CYCLIN-B1 (sc-245, Santa Cruz), CYCLIN-B2 (sc-28303, Santa Cruz) and CYCLIN-D1 (ab134175, Abcam).
Validation	All purchased antibodies have been described as suitable and independently validated for immunohistochemistry and

immunoblotting. See images and references provided on the manufacturer's websites. Custom made SOX5 antibodies were validated through correlative studies on embryonic brain tissue and with immunoblotting on SOX5 transfected cell lines. Antibodies used for FACS sorting (against GPC6, HMMR, EDNRB, EFNA5 and SLC1A5) were validated for their sub-cellular localisation and cell-type enrichment using correlative immunohistochemistry with SOX2.

Eukaryotic cell lines

Policy information about cell lines

Cell line source(s)	Mouse P19 cells were directly obtained from ATCC
Authentication	Karotyping (P19 cells) n = 40; XY, n = 40; XY Reference: McBurney MW, Rogers BJ. Isolation of male embryonal carcinoma cells and their chromosome replication patterns. Dev. Biol. 89: 503-508, 1982.
Mycoplasma contamination	Mouse P19 cells were directly obtained from ATCC and therefore considered negative for mycoplasma
Commonly misidentified lines (See ICLAC register)	no commonly misidentified cell lines were used

Animals and other organisms

Policy information about studies involving animals; ARRIVE guidelines recommended for reporting animal research

Laboratory animals	Time mated adult female CD1 and adult female C57/BlJ mice were obtained from Charles River. Adult Sox2-GFP (B6;129S-Sox2tm2Hoch/J) were obtained from the Jackson laboratory. Mice were bred and kept under pathogen free conditions at the Karolinska Institutet animal facility. Handling of animals was performed in accordance with regulations defined by FELASA. Breeding Sox2-GFP males were utilised from 10-52 weeks of age. All female C57/Bl and CD1 mice were aged from 8-12 weeks.
Wild animals	No wild animals were used in the study
Field-collected samples	No field-collected samples were used in the study.
Ethics oversight	All animal procedures and experiments were performed in accordance with Swedish animal welfare laws authorized by the Stockholm Animal Ethics Committee: Stockholm Norra Dnr N249/14.

Note that full information on the approval of the study protocol must also be provided in the manuscript.

Flow Cytometry

Plots

Confirm that:

☒ The axis labels state the marker and fluorochrome used (e.g. CD4-FITC).

☒ The axis scales are clearly visible. Include numbers along axes only for bottom left plot of group (a 'group' is an analysis of identical markers).

☒ All plots are contour plots with outliers or pseudocolor plots.

☒ A numerical value for number of cells or percentage (with statistics) is provided.

Methodology

Sample preparation	Embryonic mouse cortices were dissected and dissociated using a Miltenyi Neural Tissue Dissociation Kit (#130-092-628). Sox2-GFP embryo cortices were immunostained with specific cell-surface protein antibodies and subjected to FACS on FACSvantage/DiVa to obtain a double positive population of > 98% purity.
Instrument	BD FACSAria III and FACSvantage/DiVa
Software	BD FACSDiVa software
Cell population abundance	The antibody sorted populations ranged from 0.5% to 5% of the parent population, and at least 30000 cells were obtained.
Gating strategy	Cells were first gated for Sox2-GFP expression (P4 in Supplementary figure 4g-i) and then for Cy5 antibody signal.

☒ Tick this box to confirm that a figure exemplifying the gating strategy is provided in the Supplementary Information.
