# Supplementary material for: CYCLIN-B1/2 and -D1 act in opposition to coordinate cortical progenitor self-renewal and lineage commitment
Source: Nat Commun. 2020 Jun 9;11:2898. doi: 10.1038/s41467-020-16597-8 (PMC7283355; doi:10.1038/s41467-020-16597-8)
Supplement: Supplementary file 4 — Description of Additional Supplementary Files [file 41467_2020_16597_MOESM4_ESM.pdf]

Description of Additional Supplementary Files

File Name: Supplementary Data 1
Description: Relevant statistics of single cell sequencing.

File Name: Supplementary Data 2
Description: Genes used as input for nearest neighbour (tSNE-NN) mapping in Fig. 1 and Supplementary Fig. 1.

File Name: Supplementary Data 3
Description: Description of total RPKMs for all cells across all the genes.

File Name: Supplementary Data 4
Description: Genes differentially expressed (SCDE) between neuroblast/neuronal Infomap clusters of early and late differentiation trajectories.

File Name: Supplementary Data 5
Description: Genes included in upper-layer trajectory and deep-layer trajectory gene sets.
